# Supplementary material for: Transgene Expression Kinetics and Replication Potential of Recombinant Adenovirus Serotype 4 in a Mouse Model and its Use as a Herpes Simplex Virus Vaccine
Source: bioRxiv. 2026 May 16:2026.05.15.725395. Preprint. [Version 1] doi: 10.64898/2026.05.15.725395 (PMC13192867; doi:10.64898/2026.05.15.725395)

bioRxiv preprint doi: https://doi.org/10.64898/2026.05.15.725395; this version posted May 17, 2026. The copyright holder has placed this preprint (which was not certified by peer review) in the Public Domain. It is no longer restricted by copyright. Anyone can legally share, reuse, remix, or adapt this material for any purpose without crediting the original authors.

SUPPLEMENTAL MATERIAL:

Supplemental Fig. 1. Limit of detection calculation for selected IVIS Assay (A) Average radiance of the whole body of three uninfected BALB/c mice (1 female, 2 male) imaged either supine (n=2) or prone (n=1). Each point represents an independent imaging session. Median average radiance value (1,250 average radiance) was used to determine the limit of detection of the assay. (B) Average radiance of the whole body of two uninfected HuCD34 mice (2 female) imaged either supine (n=1) or prone (n=1). Each point represents an independent imaging session. Median average radiance value (1,900 average radiance) used to determine the limit of detection of the assay. (C) Organ-specific ex vivo background bioluminescence from uninfected mice (n=2). Median average radiance (818 average radiance) (D) Median average radiance from uninfected ex vivo lungs (434 average radiance) from uninfected mice (n=3). Dotted lines in all panels represent the limit of detection for each assay defined as twice the median average limit of detection.

Supplemental Fig. 2. Example of in vivo and ex vivo IVIS imaging of BALB/c mice 4 days after inoculation with Ad4-Luc. (A) In vivo versus ex vivo comparison of organ specific luciferase expression 4 days after intramuscular inoculation with Ad4-Luc (6.8 $x10^6$ total IFU in the right thigh). Luciferase signal is detected in the liver. (B) In vivo versus ex vivo comparison of organ specific luciferase expression four days after intranasal inoculation with Ad4-Luc (5.4 $x10^6$ total IFU). Luciferase signal is detected in the lungs.

bioRxiv preprint doi: https://doi.org/10.64898/2026.05.15.725395; this version posted May 17, 2026. The copyright holder has placed this preprint (which was not certified by peer review) in the Public Domain. It is no longer restricted by copyright. Anyone can legally share, reuse, remix, or adapt this material for any purpose without crediting the original authors.

Supplemental Fig. 3. Ad4 neutralizing antibody titers in the serum after two intranasal doses Ad4-Luc. Ad4 serum neutralization titers (50% inhibitory concentration, IC_{50}) of mice immunized with two intranasal doses of Ad4-Luc (given on day 0 and day 42) at days -2, 63 and 94. Horizontal lines indicating the group means. Dashed line is limit of detection of assay. *: $p = 0.032$; ns: not significant.

Supplemental Figure 1:

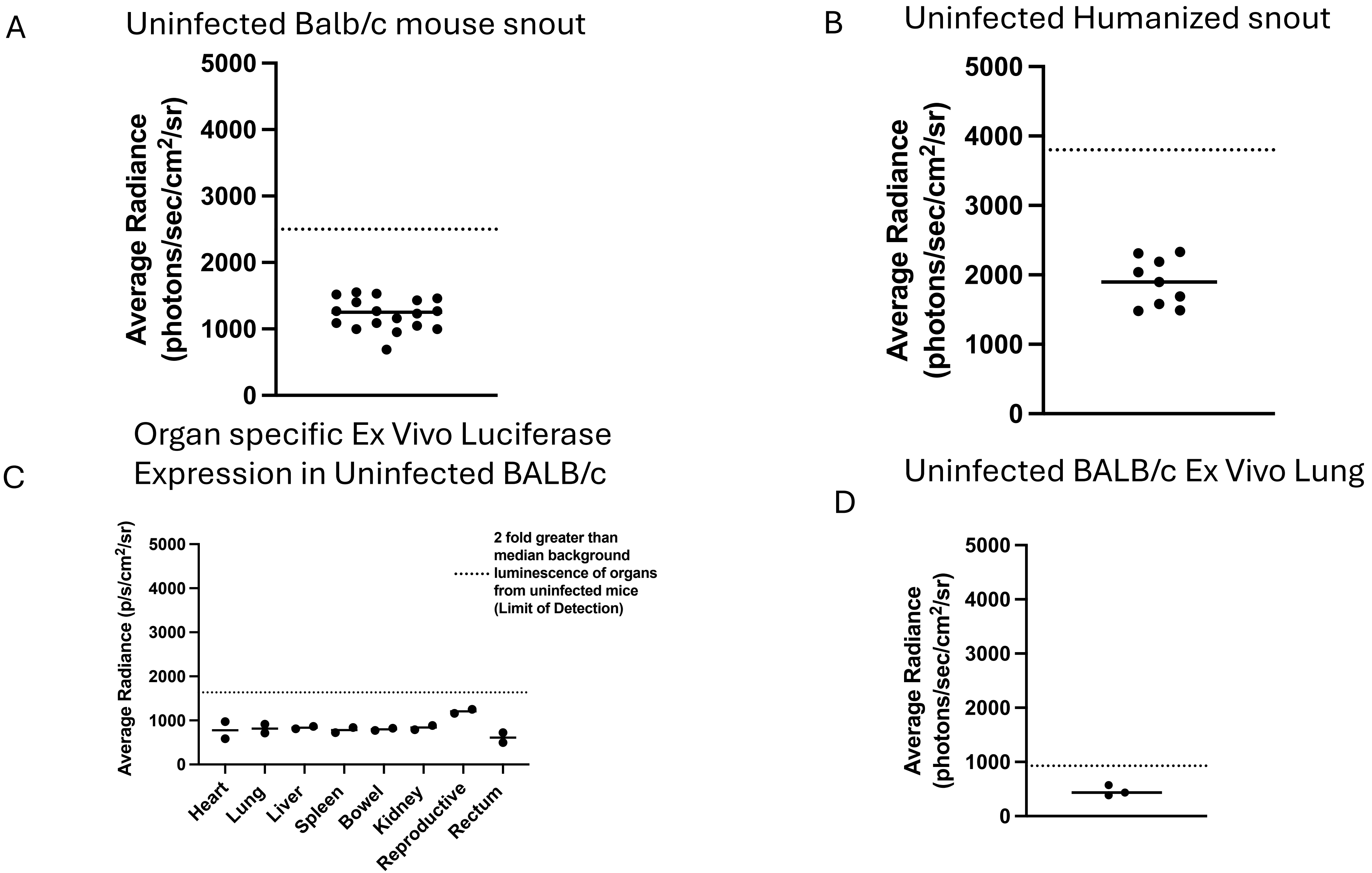

Supplemental Figure 2:

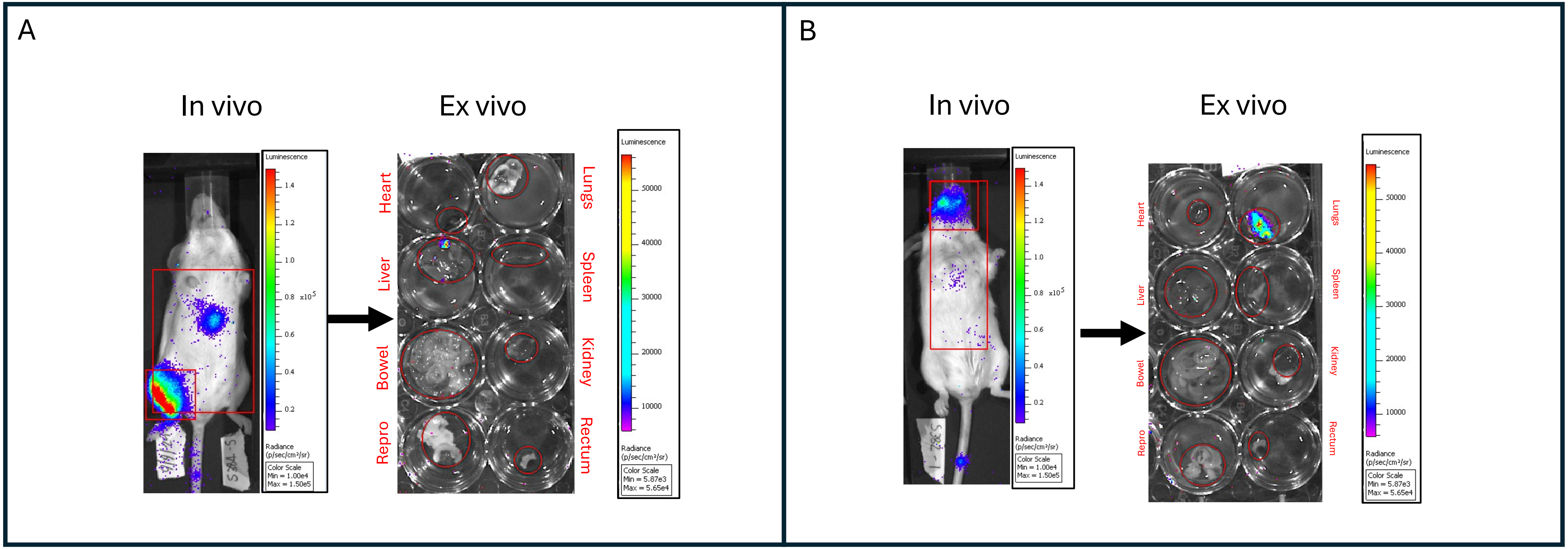

Supplemental Figure 3:

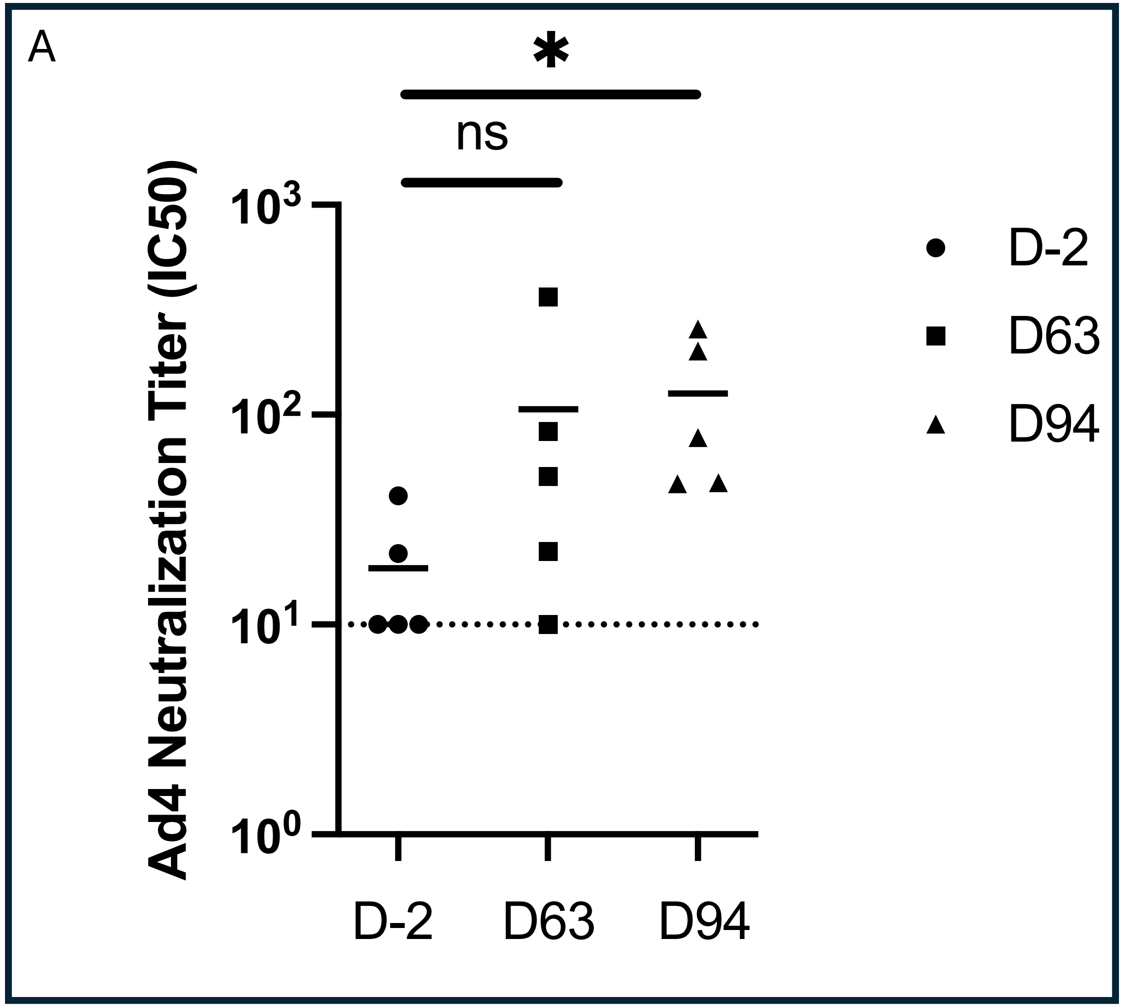

Supplement: Supplement 1 [file NIHPP2026.05.15.725395v1-supplement-1.pdf]
